# Validation of the Rainbow Model of Integrated Care Measurement Tools (RMIC-MTs) in renal care for patient and care providers

**DOI:** 10.1371/journal.pone.0222593

**Published:** 2019-09-19

**Authors:** Pim P. Valentijn, Fernando Pereira, Christina W. Sterner, Hubertus J. M. Vrijhoef, Dirk Ruwaard, Jörgen Hegbrant, Giovanni F. M. Strippoli

**Affiliations:** 1 Department of Patient and Care, Maastricht University Medical Center, Maastricht, The Netherlands; 2 Department of Health Services Research, Care and Public Health Research Institute (CAPHRI), Faculty of Health, Medicine and Life Sciences, Maastricht University, Maastricht, The Netherlands; 3 Integrated Care Evaluation, Essenburgh, Hierden, The Netherlands; 4 Strategy and Health Economics Office, Diaverum, Madrid, Spain; 5 Strategy and Health Economics Office, Diaverum, Gothenburg, Sweden; 6 Department of Family Medicine, Vrije Universiteit Brussel, Brussels, Belgium; 7 Panaxea, Amsterdam, The Netherlands; 8 Diaverum Medical Scientific Office, Diaverum Sweden AB, Lund, Sweden; 9 Sydney School of Public Health, The University of Sydney, Sydney, Australia; 10 Department of Emergency and Organ Transplantation, University of Bari, Bari, Italy; University of Florence, ITALY

## Abstract

**Introduction:**

Integrated service delivery is considered to be an essential condition for improving the management and health outcomes of people with chronic kidney disease (CKD). However, research on the assessment of integrated care by patients and care providers is hindered by the absence of brief, reliable, and valid measurement tools.

**Objective:**

The aim of this study was to develop survey instruments for healthcare professionals and patients based on the Rainbow Model of Integrated Care (RMIC), and to evaluate their psychometric properties.

**Design:**

The development process was based on the US Food and Drug Administration guidelines. This included item generation from systematic reviews of existing tools and expert opinion on clarity and content validity, involving renal care providers and chronic kidney patients. A cross-sectional, multi-centre design was used to test for internal consistency and construct validity.

**Setting:**

Outpatient clinics in a large renal network.

**Participants:**

A sample of 30.788 CKD patients, and 8.914 renal care providers.

**Methods and analysis:**

Both survey instruments were developed using previous qualitative work and published literature. A multidisciplinary expert panel assessed the face and content validity of both instruments and following a pilot study, the psychometric properties of both instruments were explored. Exploratory factor analysis with principal axis factoring and with promax rotation was used to assess the underlying dimensions of both instruments; Cronbach’s alpha was used to determine the internal constancy reliability.

**Results:**

17.512 patients (response rate: 56.9%) and 8.849 care providers (response rate: 69.5%) responded to the questionnaires. Factor analysis of the patient questionnaire yielded three internally consistent (Cronbach’s alpha > 0.7) factors: person-centeredness, clinical coordination, and professional coordination. Factor analysis of the provider questionnaire produced eight internally consistent (Cronbach’s alpha > 0.7) factors: person-centeredness, community centeredness, clinical coordination, professional coordination, organisational coordination, system coordination, technical and cultural competence. As hypothesised, care coordination patient and providers scores significantly correlated with questions about quality of care, treatment involvement, reported health, clinics’ organisational readiness, and external care coordination capacity.

**Conclusion:**

This study provides evidence for the reliability and validity of the RMIC patient and provider questionnaires as generic tools to assess the experience with or perception of integrated renal care delivery. The instruments are recommended in future applications testing test-retest reliability, convergent and predictive validity, and responsiveness.

## Introduction

The number of people with Chronic Kidney Disease (CKD) across the world is growing due to the rising rates of diabetes mellitus, obesity, hypertension and ageing population [1]. While the number of CKD patients is growing, so are the complexities of their healthcare needs. In fact, 87% of this population has two or more chronic illnesses or multimorbidity [2]. A growing number of CKD patients is in need of health services from multiple providers and units over time, and hence request the coordinated delivery of care. Yet, studies have shown that CKD patients experience multiple obstacles like communication barriers, polypharmacy, delay in referral to a nephrologist, and lack of clear delineation of responsibilities among providers throughout their care treatment trajectory [3–5]. Reasons for this suboptimal situation are rooted in the complex interplay of clinical, professional, organisational and system factors influencing access and coordination of care. Valid and reliable measurement instruments are needed to assess how these factors influence the process of care coordination, and to pin-point key areas of improvement that need to be enhanced in delivering integrated renal care practice.

Over the past decade, integrated care research has been criticised for lacking both a clear definition and for methodological problems relating its measurement including validity and reliability [6]. Integrated care has shown great overlap with related concepts such as patient-centred care, and coordinated care [7,8], which makes it difficult to measure the concept. In general, coordinated care refers to teamwork between different care providers, and patient-centred care is about involving patients in their own care. However, integrated care is considered an overarching multidimensional concept not only including the patient involvement and collaboration among care providers but also the cross-boundary cooperation between different care organisations [7,8]. The World Health Organization (WHO) has defined integrated care as health services that are managed and delivered in a way so that patients receive a continuum of preventive and curative services according to their needs over time that is coordinated across different levels (e.g. clinical, professional, organizational) of the health system [9]. A guiding influence for this definition was the Rainbow Model of Integrated Care (RMIC) identifying the four core (person-centeredness; service coordination; professional coordination; and organisational coordination) and four ancillary (community-centeredness, technical competence, cultural competence; and system context) integrated care domains [10]. Throughout this article both coordinated care and integrated care are used interchangeable and refer to as ‘integrated care’.

A recent systematic review has shown that measurement instruments assessing integrated care have significant limitations [6]. Firstly, the psychometric properties (e.g. validity, reliability etc.) of most measurement instruments are of low to moderate quality. Secondly, the majority of existing instruments contains scales to assess the person-focused care, clinical and professional coordination domains within intuitional settings, and fail to assess the full range of integrated care domains as described by the RMIC. Finally, instruments with desirable psychometric properties have too many items to be practical for use in routine practice. In sum, there is a lack of brief, reliable, and validated measurement tools for assessing the multidimensional concept of integrated care from the perspectives of patients and care providers.

Based on the RMIC, a literature review and two international Delphi studies were conducted to develop the first version of a measurement tool (MT). The RMIC-MT had 44 items for assessing healthcare providers perceptions of the delivery of integrated care in terms of the eight domains of the RMIC using a four point Likert scale (S1 Table). This preliminary version of the RMIC-MT has been tested in the Netherlands [11], Australia [12], and Singapore [13]. These studies showed that the RMIC-MT provider version was a highly relevant and easy to use instrument with good psychometric properties for the clinical coordination, cultural competence and person-centeredness scales. However, further work was needed to improve the psychometric properties of the professional coordination, organisational coordination, system coordination, technical competence and community-centeredness scales, and respondents advised to develop a RMIC-MT patient version [14]. The aim of this study was to develop the RMIC-MT for CKD patients and renal care providers. And to explore the factor structure and psychometric properties of the RMIC-MT patient and provider version.

## Methods

**Fig 1:**
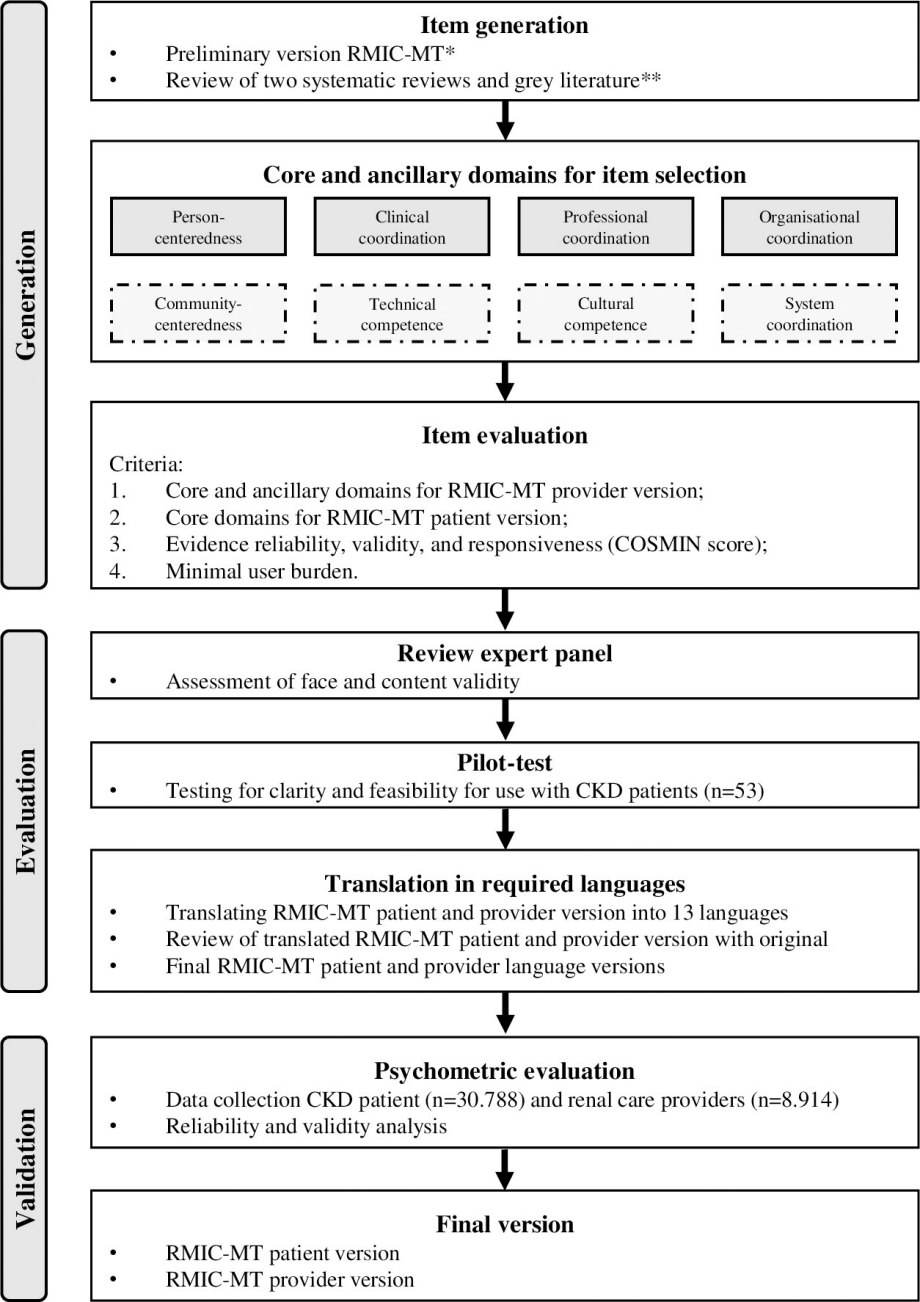
Study design. * Based on Nurjono et al. (2016) [13] and Valentijn et al. (2015) [19]. ** Based on Bautista et al. (2016) [6], Uijen et al (2012) [16], and search of grey literature [22, 35–37].

The development of the RMIC-MT patient and provider version was based on the PROM guidelines published by the US Food and Drug Administration [15], and encompassed three stages: 1) generation of items that represent the domains of the RMIC; 2) evaluation of face and content validity, clarity and feasibility; and 3) validation of the scales of the RMIC-MT patient and provider version (Fig 1).

### Instrument development

#### Item generation

The preliminary version of the RMIC-MT was used to develop an improved patient and provider version [12,13]. The development was undertaken by reviewing two systematic reviews of care coordination and integration questionnaires [6,16], and an additional search of the grey literature. One researcher (PV) reviewed all questionnaires, and generated an item pool of clinician-reported and patient-reported measures that represented the domains of the RMIC.

#### Item evaluation

Two researchers (PV, FAP) independently reviewed the item pool of the RMIC-MT patient and provider version using a standardized evaluation form. Any discrepancies were resolved through iteration and discussion. Items were considered eligible if: 1) The content of an item reflected the core and ancillary domains of the RMIC for the provider version, and only the core domains for the patient version; 2) items provide evidence for its reliability, validity and responsiveness based on the COSMIN scores reported by Uijen, et al. (2012) [16] and Bautista, et al (2016) [6]; 3) items have a minimal user burden (i.e., ≤ 9 question items and simple response categories) for patient and care providers to complete.

### Instrument evaluation

#### Assessment of face and content validity

An expert panel of seventeen persons in the UK was convened to assess the face and content validity of the RMIC-MT patient and provider version. This panel was multidisciplinary and represented the following stakeholder groups: 1) practitioners (e.g. nephrologist, nurse, or dietician); 2) managers (e.g. clinic managers, or human resource directors), and 3) service users (e.g. people with end-stage kidney disease and treated with haemodialysis). Each panel member was asked to review both instruments independently using the following criteria: 1) the clarity of the questions and instruction texts (yes or no); 2) the redundancy of the questions included (yes or no); and 3) the relevance of the questions included to measure integrated care on a four-point Likert scale ranging from (1) not relevant to (4) highly relevant. A space for comments for each question was provided and members were also asked to review the demographic questions. The first three criteria (i.e. clarity, redundancy, and relevance) were used to assess the face validity of both the RMIC-MT patient and provider version. The fourth criterion (relevance) was used to assess the content validity of both instruments. Based on the relevance score of each item, the Item Content Validity Index (I-CVI) was calculated. The I-CVI is the proportion of each individual item that received a rating of 3 or 4 by the expert panel. For each scale, a Scale Content Validity Index (S-CVI _Ave_) was calculated. This is the average of all the I-CVI’s of the individual items. An I-CVI of 0.78 and an S-CVI of 0.90 is considered to be excellent [17]. Based on these criteria, the final RMIC-MT patient and provider version were produced.

#### Testing for clarity and feasibility for use with patients

The RMIC-MT patient version was tested using a sample of 53 CKD patients selected from a collaborative network of 4 dialysis clinics in the UK. Patients were eligible to participate if they were aged over 18 years, had end-stage kidney disease, able to communicate in English, and consented to participate. The instrument was administered by a research assistant using a tablet computer. Data collection took place in June 2017. A standardized feedback form was used as a further evaluation of the RMIC-MT patient version for length, clarity, and presence of distressing questions [18]. In addition, patients were asked to identify which question, if any, they found difficult to answer, and if any of the questions had concerned or upset them. Space was provided for additional comments.

#### Translating into required languages

The English version of the RMIC-MT patient and provider version were translated to Spanish, Arabic, French, German, Hungarian, Italian, Kazakh, Lithuanian, Polish, Portuguese, Romanian, Russian, and Swedish by a translation company with the relevant linguistic background. The translated versions of the RMIC-MT’s were independently reviewed against the original English version by a country manager or lead physician and minor revisions were made to adapt to the local language and culture. No points of misunderstanding were detected. Subsequently, the translated versions of the RMIC-MT patient and provider version were administrated for the study.

### Instrument validation

#### Design

A cross-sectional study design including a convenience sample of 8.421 renal care providers (e.g. nephrologists, nurses, and management) and 30.788 CKD patients within an international collaborative network of 316 dialysis clinics in 19 countries (e.g. Argentina, Australia, Chile, France, Germany, Global, Hungary, Italy, Kazakhstan, Lithuania, New Zealand, Poland, Portugal, Romania, Russia, Saudi Arabia, Spain, Sweden, UK, and Uruguay) was used for the validation of the RMIC-MT patient and provider version. Participating clinics received a written information package consisting of an introduction letter and patient information sheet to inform the clinic managers, care providers and patients about the study’s purpose and data collection methods. The RMIC-MT’s were distributed to all clinic managers, care providers and patients in each of the participating site between 25 September-13 November 2017. All participants were asked written informed consent before enrolment in the study procedure. The RMIC-MT’s were completed online using a web-based survey platform. A forced answering procedure (i.e. respondents had to answer each question before they were allowed to proceed to the next question) was used to prevent missing answers [19]. Via clinic-specific codes assigned to each questionnaire, the response rate per clinic was checked and reported back to each dialysis clinic once a week during the data collection period.

#### Study population

In the participating clinics, care providers (i.e. nephrologist, nurse, psychologist, dietician, clinic managers) were considered eligible if they met the following criteria: 1) were actively involved in the clinical and/or administrative process of the dialysis clinic; and 2) worked at least one full month for at least 8 hours/week on their site. Care providers were excluded if they were unable or unwilling to provide informed consent. Patients were considered eligible to participate in the study if they met the following criteria: 1) aged 18 years or older; 2) end-stage kidney disease treated with haemodialysis for 90 days or longer. Patients were excluded if they: 1) were unable or unwilling to provide informed consent; 2) were unable to complete an online questionnaire even with help of carers; and 3) had a life expectancy less then 12 months.

#### Sample size calculation

The estimated minimal sample was based on the requirement of 10 subjects per item within each RMIC-MT questionnaire [20]. Given that the RMIC-MT provider questionnaire had 48 items and the patient questionnaire 24 items, the required sample size was 480 and 240. Thus, the study included a representative sample bigger than that recommend for the statistical analysis.

#### Study variables RMIC-MT provider version

The provider version assessed how renal care providers perceived the clinics ability to deliver integrated care on a five-point Likert scale (i.e. never, rarely, sometimes, often, always) on 48 items: person-centeredness (e.g. needs assessment), community-centeredness (e.g. population screening), service coordination (e.g. personal care plan), professional coordination (e.g. multidisciplinary team), organisational coordination (e.g. inter-organisational partnerships), system coordination (e.g. policy and financing), technical competence (e.g. interoperable medical records), and cultural competence (e.g. collaboration culture) [21]. Care providers were also asked to rate the overall perceived ability to coordinate care internally and externally on a 10-point scale ranging from very poor (1) to excellent (10) [19]. In addition, the adaptive reserve of the clinic was assessed using the resource and culture subscale on a five-point Likert scale derived from the work of Helfrich et al. [22]. Finally, data on type of profession were collected.

#### Study variables RMIC-MT patient version

The patient version assessed how patients experienced the integration of care on a five-point Likert scale (i.e. never, rarely, sometimes, often, always) with 24 items: person-centeredness (e.g. needs assessment), service coordination (e.g. personal care plan), professional coordination (e.g. multidisciplinary team), and organisational coordination (e.g. inter-organisational partnerships) [21]. Patients were also asked which care providers they had visited outside the dialysis clinics and how they perceived the cooperation with the dialysis clinic on a five-point Liker scale ranging from poor (1) to very good (5). In addition, patients were asked to rate the overall perceived coordination, quality and involvement of care on a 10-point scale ranging from very poor (1) to excellent (10) [19]. Finally, the following socio-demographic data were collected: age; gender; marital status; work status; and health status on a five-point Likert scale ranging from very poor (1) to very good (5) [23].

#### Statistical analysis

Data were entered, cleaned and checked before the analysis. Continues variables were expressed as mean and standard deviations. Frequencies and percentages were used for categorical variables. Distribution properties of responses to the RMIC-MT items were used to study the psychometric sensitivity. Items with a skewness (Sk) values > 3 and kurtoris (Ku) > 7, were considered to have psychometric sensitivity issues [24]. Items with a floor or ceiling effect of > 75% respondents were considered problematic and deleted [25].

Exploratory factor analysis with principal axis factoring extraction method and promax (oblique) rotation were used to assess the underlying structure of the RMIC-MT patient and provider questionnaires [26]. Barlett’s test of sphericity and Kaiser-Meyer-Olkin (KMO) measurement of sampling adequacy were used to determine if the requirements for a factor analysis were met [27]. The number of factors to consider were determined by considering the eigenvalues (>1), scree plot, and interpretability of the factor. More importantly, the factors retained had to be guided theoretically [26]. Names were given for each identified factor based on the domains of the RMIC. Items that crossed loaded on more than 1 factor were placed with the factor that was most closely related conceptually. Items with poor factor loadings (< 0.6) were removed from the final questionnaires [26]. Additionally, a structural equation model with maximum likelihood was used to evaluate the explorative factor analysis model fit by using the standard fit indices: root-mean-square error of approximation RMSEA (≤0.06, 90% CI ≤0.06), standardized root-mean-square residual (SRMR) (≤0.08), comparative fit index (CFI) (≥0.95), Tucker-Lewis index (TLI) (≥0.95), and the chi-square/df ratio less then 3 [26].

The internal consistency was assessed using item-total correlations and Cronbach’s alpha. Item-total correlations assess the overall correlation between items within a scale, and should be ≥ 0.4. A Cronbach’s alpha of ≥ 0.70 was considered acceptable for a scale to be sufficiently reliable [28,29]. Pearson correlation coefficients (r) were calculated to assess whether each item was in the right subscale by correlating items with the subscale means. Items that correlated more highly on subscales other than the one to which it was assigned were eliminated [30,31].

Construct validity was assessed by calculating Pearson’s correlations between the integrated care scale scores and two overall perceived coordination questions within the RMIC-MT patient and provider version. Moderately positive associations (≥0.4) between integrated care and these correlates would indicate good construct validity [32]. For patients, the following hypotheses were tested based on previous studies [33]: 1) patients who have a better coordinated care experience are more satisfied with (a) quality of care, and (b) treatment involvement; 2) each instruments subscale aims to measure coordinated care experience and are therefore positively and significantly correlated with other subscales; and 3) patients who report their health as good are more positive regarding the care coordination experience than patients who report their health as poor. For care providers, the following hypotheses were tested: 1) care providers who indicate a better care coordination ability are more satisfied with (a) the internal adaptive reserve, and (b) external care coordination ability; and 2) each instruments subscale aims to measure the clinics coordinated care ability and are therefore positively and significantly correlated with other subscales. P-values <0.05 were considered statistically significant. All statistical analyses were done using SPSS version 23.0 (IBM SPSS Statistics, 2015), and AMOS statistical package version 23.

### Ethics

Ethical approval for this study was waived by the Independent Review Board Nijmegen (IRBN) [34] because the study was considered noninterventional. The committee concluded that this study was conducted in accordance with the ethical principles that have their origin in the Declaration of Helsinki and that are consistent with good clinical practice (GCP). Written informed consent was obtained from each participant before collecting data.

## Results

### Instrument development

Based on two systematic reviews [6,16] and four additional publications [22,35–37], we identified 234 integrated care instruments, of which 58 were considered potentially eligible. Of these 58 instruments, we used the items of six instruments [25,35,37–40] to improve the professional, organisational, system and technical competence scales of the existing RMIC-MT provider version (73 items), and the items of four instruments [31,41–43] were selected to construct RMIC-MT patient version (24 items). After one revision round the RMIC-MT provider version consisted of 50 items grouped into eight domains, and the RMIC-MT patient version consisted of 28 items grouped into four domains.

### Instrument evaluation

#### Face and content validity

Six practitioners, five managers, and one CKD patient participated (response rate = 71%) to review the RMIC-MT provider version, and four practitioners, three managers, and two CKD patients (response rate = 53%) to review the RMIC-MT patients’ version. The face validity scores of both the RMIC-MT patient and provider version are tabulated in Table 1. The most overlapping or redundant questions were found for the professional coordination scale for both the patient and provider version. In addition, the participants considered the cultural competence scale of the provider version redundant. Based on the qualitative comments made by the participants, items of the community centeredness, system coordination, and technical competence scales of the provider version were revised. In addition, questions of clinical coordination and professional coordination scales of the patient version were changed based on the qualitative comments made by the participants. Twenty-four of the 25 items of the RMIC-MT patient version had an excellent content validity (I-CVI ≥ 0.78), and one item had a fair content validity (I-CVI < 0.78). The average scale content validity (S-CVI _Ave_) for the RMIC-MT provider version ranged from 0.68 for the organisational integration scale and 0.97 for the person-focused care scale (Table 1). The S-CVI _Ave_ for the entire RMIC-MT provider version was 0.85. The average scale content validity (S-CVI _Ave_) for the RMIC-MT patient version ranged from 0.75 for the organisational coordination scale and 0.96 for the person-centeredness scale (Table 1). The S-CVI _Ave_ for the entire consumer questionnaire was 0.87.

**Table 1:** Face and content validity RMIC-MT patient and provider version.

Scale	Patient version (n = 9)				Provider version (n = 12)			
	No. of items	Clarity (% yes)	Redundancy (% yes)	S-CVI _Ave_	No. of items	Clarity (% yes)	Redundancy (% yes)	S-CVI _Ave_
Person-centeredness	6	98	7	0.96	5	88	7	0.97
Community centeredness	NS	NS	NS	NS	4	88	8	0.79
Clinical coordination	8	97	6	0.87	7	95	2	0.94
Professional coordination	8	78	17	0.89	7	92	10	0.93
Organisational coordination	6	96	2	0.74	5	87	8	0.68
System coordination	NS	NS	NS	NS	3	89	8	0.86
Technical competence	NS	NS	NS	NS	11	83	7	0.88
Cultural competence	NS	NS	NS	NS	8	93	11	0.77

Abbreviations: S-CVIave, Average Scale Content Validity Index; NS, not stated.

#### Clarity and feasibility

Fifty-three CKD patients participated in the pilot study using the RMIC-MT patient version.

The average age of the patients in the sample was 67 years (SD 15) with 55% male and 45% female. The majority of the patients were retired (72%) and their care was coordinated by their nephrologist (89%) (S2 Table). The mean completion time for the RMIC-MT patient was 9 minutes (SD: 2.7), with a range of 5 to 20 minutes. Overall, 13% (n = 7) of patients had help completing the questionnaire (S3 Table). In addition, only one patient considered one question difficult, and none of the patients considered the questions upsetting. Based on the results of the feasibility study, it was decided that no changes to the instrument were indicated.

### Instrument validation

#### Data collection

A total of 17.512 CKD patients (56.9% response rate) and 5.849 care providers (69.5% response rate) completed the questionnaires. There were no missing values in the data because all items were set as required (see details in method section). The mean age of the patients was 61.9 (SD: 15.5, range 4–118) years. A small majority of the patients were male (51.9%, n = 9084) and retired (52.3%, n = 9164). The majority of care providers were represented by nurses (54.4%, n = 3179). The demographic and characteristics of the participants are listed in Table 2.

**Table 2:** Characteristics of the study participants.

Variable	Value
***CKD patients***	***17512***
Gender, n (%)	
Male	9084 (51.9)
Female	7009 (40)
Age (years), mean (SD), range	61.86 (15.5) 4–118
Marital status, n (%)	
Married	7009 (40)
Single	9084 (51.9)
Self-reported health status, n (%)	
Very good	3229 (18.5)
Good	7398 (42.2)
Fair	4115 (23.5)
Poor	720 (4.1)
Very Poor	152 (0.9)
Work status, n (%)	
Employed	1515 (8.7)
Unemployed and looking for work)	490 (2.8)
In full time education	154 (0.9)
Unable to work due to long term sickness	2248 (12.8)
Looking after family	1896 (10.8)
Retired	9164 (52.3)
Other	834 (4.8)
***Renal care providers***	***5849***
Job position, n (%)	
Nephrologist	578 (9.9)
Nurse	3179 (54.4)
Dietician	69 (1.2)
Psychologist	38 (0.6)
Social worker	92 (1.6)
Endocrinologist	4 (0.1)
Cardiologist	1 (0.0)
Management & administration	288 (4.9)
Other	1458 (24.9)

#### Item score distribution

No items of the RMIC-MT patient and provider version presented severe floor, ceiling, Sk and Ku values, which indicates the adequate psychometric sensitivity of the items. S4 Table presents the summary measures of the items of the RMIC-MT patient and provider version.

#### Factor analysis

The KMO test of sample adequacy (0.97) and the Barlett’s test of sphericity (*p* < 0.0001) indicated that a factor analysis for the RMIC-MT patient version was appropriate. The analysis yielded two factors with eigenvalues > 1, which accounted for 51.2% of the variance. And two factors with eigenvalues < 1, accounted for 5.8% of the variance were also included because they were interpretable based on the RMIC. Hence a four-factor solution was chosen. Factor 1 was named ‘clinical coordination’ (6 items, 46.6% of variance), factor 2 ‘professional coordination’ (6 items, 4.6% variance), factor 3 ‘organisational coordination’ (6 items, 3.5% variance), and factor 4 ‘person-centeredness’ (2 items, 2.3% variance). In this solution, 8 items were omitted (i.e. item 8, 7, 11, 12, 13, 15, 22, and 19) because the factor loadings were below < 0.6, see Table 3. Regarding model fit (4-factors, 16 items), the following tests of significance and goodness-of-fit measures were obtained: x2 (98) = 5587.7; p <0.0001; x2/df = 57 | CFI = .97| TLI = 0.97| RMSEA (HI90) = .057(0.058) / SRMR = .033. Thus, the 4-factor explorative factor model showed an acceptable fit, except for the chi-square/df ratio.

**Table 3:** Factor analysis RMIC-MT patient version (n = 17,512).

		Rotated factor loadings*			
Item No.	Content	1. Clinical coordination	2. Professional coordination	3. Organisational coordination	3.Person-centeredness
2	Listening	**0.93**			
3	Preference integration	**0.91**			
5	Questioning	**0.87**			
4	Communicating	**0.85**			
1	Explaining	**0.80**			
6	Shared decision-making	**0.78**			
8	Treatment longitudinally	0.35			
7	Medical continuity	0.34			
11	Needs assessment	0.33			
17	Interdisciplinary information continuity		**0.99**		
18	Interdisciplinary treatment continuity		**0.88**		
16	Interdisciplinary contact		**0.63**		
14	Interdisciplinary coordination		**0.61**		
12	Interdisciplinary communication		**0.45**		
13	Interdisciplinary collaboration	0.30	**0.42**		
15	Interdisciplinary fragmentation				
23	Time management			**0.79**	
20	Appointments			**0.66**	
21	Results			**0.66**	
24	Accessibility			**0.63**	
22	Multidisciplinary team			**0.57**	
19	Accessibility			**0.47**	
9	Family circumstances				**0.87**
10	Social circumstances	** **	** **	** **	**0.85**
Eigenvalues		**11.19**	**1.01**	**0.85**	**0.54**
% of variance		**46.60**	**4.57**	**3.52**	**2.27**

* Factor loadings above 0.3 are reported.

In addition, the KMO test of sampling adequacy (0.95) and Barlett’s test of sphericity (*p* < 0.0001) indicated that the criteria for a factor analysis were also met for the RMIC-MT provider version. A nine-factor solution was obtained with a total of 48 items, which explained 57.6% of the variance. The analysis yielded six factors with eigenvalues > 1, which accounted for 53.2% of the variance. And three factors with eigenvalues < 1, accounted for 4,4% of the variance were also included because they were interpretable based on the RMIC.

Hence, a nine-factor solution was obtained. Factor 1 was named ‘cultural competence’ (8 items, 29.7% of variance), factor 2 ‘person-centeredness’ (5 items, 6.7% of variance), factor 3 ‘technical competence’ (5 items, 6.3% of variance), factor 4 ‘professional coordination’ (5 items, 5.2% of variance), factor 5 ‘clinical coordination’ (7 items, 3.0% of variance), factor 6 ‘Triple Aim’ (5 items, 2.3% of variance), factor 7 ‘clinical coordination’ (6 items, 1.7% of variance), factor 8 ‘system coordination’ (3 items, 1.4% of variance) and factor 9 ‘community centeredness’ (4 items, 1.3% of variance). In this solution, 12 items were omitted (i.e. item 47, 46, 48, 42, 36, 13, 14, 16, 15, 24, 21, and 2) because the factor loadings were below < 0.6, see Table 4. Regarding model fit (9-factors, 36 items), the following tests of significance and goodness-of-fit measures were obtained: x^2^ (558) = 6100.5.7; p <0.0001; x2/df = 10.9 | CFI = .96| TLI = 0.96| RMSEA (HI90) = .041(0,041) / SRMR = .026. In short, the 9-factor model showed an acceptable fit, except for the chi-square/df ratio.

**Table 4:** Factor analysis RMIC-MT provider version (n = 6,052).

Item No.	Content	Rotated factor loadings*								
		1. Cultural competence	2. Person-centeredness	3. Technical competence	4. Professional coordination	5. Clinical coordination	6. Triple Aim	7. Organisational coordination	8. System coordination	9. Community centeredness
45	Support	**0.91**								
43	Teamwork	**0.88**								
44	Respect	**0.87**								
41	Fellowship	**0.85**								
47	Learning	0.58								
46	Safety	0.53								
48	Collaboration procedures	0.52								
42	Staffing	0.50								
2	Listening		**0.92**							
1	Interpersonal trust		**0.88**							
5	Questioning		**0.78**							
4	Preference integration		**0.74**							
3	Social circumstances		**0.71**							
38	Interoperable EHRs			**0.93**						
37	Interoperable IT tools			**0.77**						
39	Data integration			**0.71**						
40	Outcome transparency			**0.66**						
36	EHRs			0.42						
18	Interdisciplinary fragmentation				**0.79**					
23	Interdisciplinary teamwork				**0.79**					
22	Interdisciplinary follow-up				**0.78**					
17	Interdisciplinary communication				**0.73**					
19	Interdisciplinary coordination				**0.70**					
11	Follow-up of care					**0.79**				
10	Case management					**0.71**				
12	Shared decision-making					**0.68**				
13	Shared care plans					0.58				
14	Quality procedures					0.49				
16	Medical continuity					0.46				
15	Multidisciplinary team					0.31				
34	Monitoring & follow-up						**0.80**			
33	Quality objectives						**0.77**			
31	Needs assessment						**0.72**			
35	Outcome assessment						**0.71**			
32	Experience assessment						**0.70**			
26	Inter-organisational resources							**0.90**		
27	Inter-organisational staff							**0.80**		
25	Inter-organisational coordination							**0.78**		
24	Inter-organisational objectives							0.52		
21	Interdisicplinary collaboration							0.33		
20	Information exchange							0.33		
29	Interdisicplinary incentives								**0.97**	
30	Care coordination incentives								**0.86**	
28	Inter-organisational incentives								**0.79**	
7	Health promotion									**0.81**
9	Population needs									**0.79**
6	Community partnership									**0.68**
8	Community collaboration	** **	** **	** **	** **	** **	** **	** **	** **	**0.67**
Eigenvalues		14.25	3.21	3.02	2.49	1.45	1.12	0.82	0.66	0.63
% of variance		29.68	6.68	6.30	5.18	3.02	2.33	1.70	1.37	1.30

* Factor loadings above 0.3 are reported.

#### Internal consistency

Internal consistency analysis showed that reliability assumptions were adequately met for all four scales of the RMIC-MT patient version. Item-total correlations exceeded 0.4 for all items, see S5 Table. Cronbach’s alpha ranged from 0.84 for the organisational coordination scale to 0.93 for the clinical coordination scale, see Table 5. Inspection of the correlation matrices revealed that all individual items highly correlated with their respective subscale then with the competing scales. Thus, the final RMIC-MT patient version is a reliable scale (alpha 0.94) comprising 16 patient experience items.

**Table 5:** Descriptive statistics and internal consistency of the RMIC-MT patient and provider version.

Scale	Patient version (n = 17,512)				Provider version (n = 5,849)			
	No. of items	Ceiling effect (n, %)	Mean, SD	Cronbach's Alpha	No. of items	Ceiling effect (n, %)	Mean, SD	Cronbach's Alpha
Person-centeredness	2	4719 (26.9)	3.96 (0.89)	0.87	5	1626 (27.8)	4.32 (0.67)	0.90
Community centeredness	NS	NS	NS	NS	4	1108 (18.9)	4.00 (0.79)	0.89
Clinical coordination	6	5684 (32.5)	4.37 (0.58)	0.93	3	2262 (37.4)	4.35 (0.67)	0.77
Professional coordination	4	4282 (24.5)	4.18 (0.66)	0.86	5	284 (4.9)	3.42 (0.83)	0.87
System coordination	NS	NS	NS	NS	3	629 (10.4)	3.61 (0.78)	0.92
Technical competence	NS	NS	NS	NS	4	569 (9.4)	3.41 (0.91)	0.84
Organisational coordination	4	5833 (33.3)	4.37 (0.59)	0.84	3	733 (12,2)	3.63 (0.82)	0.85
Cultural competence	NS	NS	NS	NS	4	1900 (31.4)	4.26 (0.80)	0.90
Triple Aim	NS	NS	NS	NS	5	2061 (34.1)	4.37 (0.68)	0,90
Overall care coordination	16	2698 (15.4)	4.27 (0.54)	0.94	36	69 (1.1)	3.95 (0.49)	0.93

Abbreviations: NS, not stated.

The internal consistency analysis also showed that the nine domains of the RMIC-MT provider version could be reliably measured. Item-total correlations exceeded 0.4 for all items, see S6 Table. The Cronbach’s alpha for the RMCI-MT provider version ranged from 0.90 for the system coordination, cultural competence, person-centeredness, and Triple Aim scale to 0.84 for the technical competence scale, see Table 5. Item scale correlations showed that all individual items highly correlated with their respective subscale compared with the competing scales. Hence, the RMIC-MT provider version is also a reliable instrument (alpha 0.94) of 36 items to measure integrated care.

#### Construct validity

All patient related hypotheses could be accepted, which confirms the construct validity of the RMIC-MT patient version. Patients who experienced better care coordination were more satisfied with the quality (*r* = 0.51, *P* <0.01) and treatment involvement (*r* = 0.40, *P* < 0.01), see Table 6. All subscales of the RMIC-MT patient version were strongly and significantly (*P* < 0.01) correlated with each other (*r* = 0.46–0.89). Finally, patients in poorer health experienced significantly (*P* < 0.01) poorer care coordination (*r* = 0.22).

**Table 6:** Correlation between scale scores RMIC-MT patient version (n = 13,191).

Variable	1	2	3	4	5	6	7	8
1. Clinical coordination	1							
2. Professional coordination	.641**	1						
3. Organisational coordination	.683**	.667**	1					
4. Person-centeredness	.507**	.559**	.458**	1				
5. Overall care coordination	.891**	.861**	.847**	.707**	1			
6. Quality of care	.477**	.429**	.447**	.319**	.512**	1		
7. Treatment involvement	.366**	.355**	.327**	.278**	.403**	.563**	1	
8. Reported health	.191**	.187**	.188**	.162**	.220**	.200**	.164**	1

** P <0.01

In addition, care providers who indicated a better care coordination ability were more satisfied about the adaptive reserve (*r* = 0.71, *P* <0.01) and external care coordination capacity (*r* = 0.59, *P* <0.01) of their clinic, see Table 7. All subscales of the RMIC-MT patient version were positively and significantly (*P* < 0.01) correlated with each other (*r* = 0.09–0.66). The strongest correlations were observed between ‘community-centeredness’ and ‘person-centeredness’ (*r* = 0.66, *P* <0.01), ‘system coordination’ and ‘organisational coordination’ (*r* = 0.63, *P* <0.01), and ‘triple aim’ and ‘clinical coordination’ (*r* = 0.62, *P*<0.01). Especially the perceived professional care coordination ability has small levels of correlation with the other scale scores (*r* = 0.09–0,24). Again, all provider related hypotheses could be accepted, which confirms the construct validity of the RMIC-MT provider version.

**Table 7:** Correlation between scale scores RMIC-MT provider version (n = 5,849).

Variable	1	2	3	4	5	6	7	8	9	10	11	12
1. Cultural competence	1											
2. Person-centeredness	.330**	1										
3. Technical competence	.246**	.147**	1									
4. Clinical coordination	.423**	.402**	.268**	1								
5. Professional coordination	.195**	.154**	.090**	.235**	1							
6. Triple Aim	.556**	.406**	.309**	.619**	.273**	1						
7. Organisational coordination	.367**	.322**	.492**	.393**	.175**	.452**	1					
8. System coordination	.340**	.289**	.474**	.354**	.180**	.394**	.630**	1				
9. Community centeredness	.323**	.658**	.270**	.404**	.144**	.380**	.459**	.406**	1			
10. Overall care coordination	.650**	.641**	.570**	.673**	.472**	.753**	.707**	.666**	.688**	1		
11. Adaptive reserve	.658**	.389**	.361**	.472**	.235**	.605**	.530**	.494**	.441**	.712**	1	
12. External care coordination	.460**	.260**	.378**	.388**	.269**	.451**	.455**	.426**	.336**	.585**	.565**	1

** P <0.01

## Discussion

### Principle findings

The RMIC-MT patient and provider version showed excellent face and content validity using an expert panel. The clarity and feasibility of the RMIC-MT patient was assessed by 53 CKD patients in a pilot study. Patients indicated that the survey instrument was easily understood and completed. The factor structure and psychometric properties of the RMIC-MT patient and provider survey instruments were tested in a cohort of 30,788 CKD patients and 8,421 renal care providers. Statistical analysis indicated that the internal consistency, reliability, and construct validity for both the RMIC-MT patient (16 items, 4 subscales) and provider version (36 items, 9 subscales) were good. The proposed models also passed the majority of goodness-to-fit test by using structural equation modelling. This suggests that the RMIC-MT is a valuable psychometric tool for evaluating care coordination as perceived by patients and care providers. Given the small number of items the utility is high to assess care coordination in routine practice.

### Comparison with other studies

The factor analysis of the RMIC-MT patient version leads us to conclude that CKD patients do differentiate between distinct, complementary domains of integrated care (i.e. person-centred care, clinical coordination, professional coordination, and organisational coordination) as described by the RMIC [10]. Yet, the organisational coordination, system coordination and community-centeredness domains did not met the eigenvalue criteria of >1 within the RMIC-MT provider version. Possibly, this multi-dimensionality was not pronounced because the organisational coordination, system coordination and community-centeredness activities are not closely related to the clinical encounter of the dialysis clinics. In addition, previous studies have also shown that care providers find it difficult to differentiate between the organisational and system domains of integrated care [44].

Most of the variance of the RMIC-MT patient version was explained by the clinical coordination domain, which has also been accentuated by other measurement tools [6]. The organisational coordination, and person-centeredness domain of the RMIC-MT patient version did not meet the eigenvalue criteria of >1. This might be due to the fact that there are (cultural) differences between the organisational and person-centeredness experiences across countries and dialysis clinics. The majority of the items hypothesised to belong to the person-centeredness domain were absorbed by the clinical coordination or did not meet the inclusion criteria. It is noteworthy that the clinical coordination domain taps on aspects related to knowing and respecting the patients’ values, which is a critical aspect to tailor the care coordination process [45]. Studies have shown that physician recognition and advance knowledge of patients’ needs can have a positive effect on patient outcomes [46].

The RMIC-MT provider version yielded nine domains reflecting all the hypothesised domains of the RMIC (i.e. person-centeredness, community centeredness, clinical coordination, professional coordination, organisational coordination, system coordination, technical, cultural competence, and Triple Aim outcomes). The majority of care coordination tools that have been developed are restricted, and only measure aspects of the clinical and professional care coordination process [6,16]. Most of the variance of RMIC-MT provider version was explained by cultural competence. This finding indicates the importance of normative trust mechanisms in the care coordination process, which has not been accentuated in previous care coordination tools [6,16]. The person-centeredness and clinical coordination domain highlights the fact that delivering integrated care is a participatory process of co-creation between care providers and patients [47].

Compared to the RMIC-MT patient version, the clinical coordination domain of the RMIC-MT provider version explained a relatively small proportion of the variance. This might suggest that care providers consider the enabling ‘backstage process’ of integrated care delivery (e.g. cultural and technical competence) more important than the domains more closely related to the clinical encounter (e.g. clinical and professional coordination) [44]. Finally, the community-centeredness domain highlights the importance of population-orientation as a guiding principle of integrated care, which has not been considered as an aspect of integrated care in previous questionnaires [6,16].

### Strengths and limitations

The strength of the RMIC-MT patient and provider version is its thoroughly development and validation process. Face and content validity are supported by using a conceptual model, results from previous validation studies, literature reviews, and multidisciplinary expert panel in the development of the RMIC-MTs questionnaires. Study results showed that both instruments had excellent face and content validity (S-CVI _avg_ > 0.78). Usability of the RMIC-MT patient version was carefully pre-tested following the method as described by Aaronson et al. (2011) [18]. Factor analysis and good levels of internal consistencies (Cronbach alpha > 0.70) of the RMIC-MT patient and provider version subscales provide evidence of reliable and valid questionnaires. Especially the RMIC-MT patient version has good evidence for construct validity with the patient related hypotheses largely being met. The significant relationship between patient care coordination scores and quality, treatment involvement, and health follows previous findings [33,48–54]. To further establish construct validity in future research, it would be interesting to test whether patient care coordination scores correlate with provider care coordination scores at dialysis clinic level. With the exception of the professional coordination and technical competence subscales, the RMIC-MT provider version had also good evidence for construct validity. In the comparison of scale scores, those measuring the organisational aspects of integration (i.e. organisational and Triple Aim) had the highest levels of correlation. We found support that for the hypotheses that a clinics adaptive reserve is a prerequisite for a better care coordination process, which is an important finding [22]. The sample size and response rates of the participants were high, which strengthens our results for the CKD population and general applicability of the RMIC-MTs. Since the RMIC-MTs were developed using a non-disease specific approach, the instruments can probably be used in other settings and other patient groups, although applicability should be assessed before using it in other settings.

However, there are a number of study limitations. First, the limited number of CKD patients participating in the expert panel is cause of concern for the content validity of the RMIC-MT patient version. Future studies should explore in more details the content validity of the RMIC-MT patient version among a larger group of patients using the I-CVI. Second, we did not use a back-translation process for ensuring linguistical validation. Although all translations were independently reviewed by bilingual experts, future research is needed on the conceptual and cultural equivalence of non-English versions of the RMIC-MT patient and provider versions. Third, while validity of the RMIC-MTs was addressed in the current study, more research is needed to assess the test-retest reliability, responsiveness to change, and construct validity against external criteria (e.g. satisfaction, quality of care, access of care) [55]. Testing the reliability (test-retest), responsiveness and construct validity of the RMIC-MTs is already planned for using a longitudinal evaluation design. Future studies should also explore how the RMIC-MTs scale scores relate to relevant patient-reported outcomes, thus establishing convergent and predictive validity. In addition, future studies should assess the discriminant validity of the RMIC-MTs, as it is likely that the care coordination process differs between clinics and care systems [56]. A fourth limitation is that the sample was chosen by convenience which may not be representative of the general CKD population and renal care providers. Although various healthcare professionals were included, the majority were nurses and nephrologists. In addition, only stage 4–5 CKD patients were included. Furthermore, the entire sample was obtained from a large renal care network within 19 countries. Hence, application of the findings to all CKD patients and renal care providers within the countries and beyond is limited. Fifth, the present study found that care providers were more critical about the clinics integrated care ability as compared with the overall patient integrated care experience. This raises the question whether the patient scores accurately reflect a very positive integrated care experience or a measurement variability limitation. To address this concern, we used an agree-to-disagree Likert scale, which is considered to generate the greatest variability in patient-based measures [57]. In addition, several scholars have shown that patients with a threatening chronic disease like CKD are reluctant to criticise their physician in terms of delivering fragmented care [58–60]. This theory requires further research regarding the discriminant validity of the RMIC-MT patient version between countries and dialysis clinics. Finally, the scales of the RMIC-MTs are meant to standardize the measurement of integrated care across different settings as patient groups. Accordingly, it is worth cross-validating the results of the present study in different patient groups and settings.

### Implications for practice

The RMIC-MT’s are valuable instruments to assess the care coordination process and can accordingly be adopted for improving the integrated delivery of renal care. Both instruments can be easily administered to care providers and patients, taking respectively 10 to 20 minutes to complete. The RMIC-MT’s can be used as a pre/post intervention measure by policymakers and commissioners to assess the impact of an integrated care programme, and by health administrators as an ongoing performance assessment tool to focus on key aspects of integrated service delivery across teams and organisations. For example, tailored information on dialysis clinics performance gives insight into the clinic’s care coordination strengths and weaknesses through their patients and care providers eyes. Evidence has shown that this internal feedback appears to be an incentive for quality improvement [61,62]. In addition, the RMIC-MT’s can be used for benchmark purposes, by distinguishing ‘strong’ from ‘weak’ performing dialysis clinics on care coordination experience and ability. Hence, we see several potential applications of the RMIC-MT’s in research, performance assessment, continuing education, and evaluation.

## Conclusion

In conclusion, this study provides evidence for the factor structure and psychometric properties of the RMIC patient and provider questionnaires as generic tools to measure integrated renal care. Both instruments serve as a useful tool for assessing integrated care through the eyes of CKD patients and renal care providers. The instruments are recommended in future applications testing test-retest reliability, convergent and predictive validity, and responsiveness.

## Supporting information

S1 Table Original RMIC-MT for care providers (44 items). (DOCX)

S2 Table Demographic characteristics of the CKD patients participating in pilot study (n = 53). (DOCX)

S3 Table Clarity and feasibility of the RMIC-MT patient version (n = 53). (DOCX)

S4 Table Summary measures of the items of the RMIC-MT patient and provider version. (DOCX)

S5 Table Descriptive statistics and internal consistency RMIC-MT patient version. (DOCX)

S6 Table Descriptive statistics and internal consistency RMIC-MT provider version. (DOCX)
