# Supplementary material for: Validation of the Rainbow Model of Integrated Care Measurement Tools (RMIC-MTs) in renal care for patient and care providers
Source: PLoS One. 2019 Sep 19;14(9):e0222593. doi: 10.1371/journal.pone.0222593 (PMC6752779; doi:10.1371/journal.pone.0222593)
Supplement: S1 Table — (DOCX) [file pone.0222593.s001.docx]

# Supplemental Table 1: Original RMIC-MT for care providers (44 items)

| **Scale** | **Example** | **No. of items** | **Response options** |
| --- | --- | --- | --- |
| Person-centred care | I.e. interventions are used to promote clients' self-care ability | 5 | Never (1) – All the time (4) |
| Community-centred care | I.e. population needs are included in the objectives of the partnership | 4 | Never (1) – All the time (4) |
| Service coordination | I.e. professionals have agreements on the referral and transfers (follow-up) of clients | 5 | Never (1) – All the time (4) |
| Professional coordination | I.e. professionals use multidisciplinary guidelines and protocols | 6 | Never (1) – All the time (4) |
| Organisational coordination | I.e. interest of the organizations involved are considered | 6 | Never (1) – All the time (4) |
| System coordination | I.e. the partnership is hampered by the rules and/or policies set by the ministries (e.g. Ministry of health) | 5 | Never (1) – All the time (4) |
| Technical competence | I.e. incentives are used to improve teamwork, coordination and continuity of care among professionals | 7 | Never (1) – All the time (4) |
| Cultural competence | I.e. activities are undertaken to better understand other organizational cultures | 6 | Never (1) – All the time (4) |

Based on Valentijn et al. (2015) [1]

# Reference

1. Valentijn PP, Vrijhoef HJ, Ruwaard D, Boesveld I, Arends RY, Bruijnzeels MA. Towards an international taxonomy of integrated primary care: a Delphi consensus approach. BMC Fam Pract. 2015;16: 64-015-0278-x.
