# Supplementary material for: Validation of the Rainbow Model of Integrated Care Measurement Tools (RMIC-MTs) in renal care for patient and care providers
Source: PLoS One. 2019 Sep 19;14(9):e0222593. doi: 10.1371/journal.pone.0222593 (PMC6752779; doi:10.1371/journal.pone.0222593)
Supplement: S2 Table — (DOCX) [file pone.0222593.s002.docx]

# Supplemental Table 2: Demographic characteristics of the CKD patients participating in pilot study (n=53)

| **Characteristic** |  |
| --- | --- |
| Gender, n (%) |  |
| Male | 29 (55) |
| Female | 24 (45) |
| Age (years), mean (SD), range | 67 (15.04), 18-89 |
| Marital status, n (%) |  |
| Married | 26 (49) |
| Single | 10 (19) |
| Divorced | 9 (17) |
| Widow | 8 (15) |
| Work status, n (%) |  |
| Employed | 2 (4) |
| Unable to work | 11 (21) |
| Looking after family | 1 (2) |
| Retired | 38 (72) |
| Self-reported health status, n (%) |  |
| Very good | 2 (4) |
| Good | 23 (43) |
| Fair | 22 (42) |
| Poor | 6 (11) |
| Very poor | NS |
| Renal care coordinator |  |
| Nephrologist | 47 (89) |
| Nurse | 6 (11) |
| Visiting care providers outside the clinic |  |
| Yes | 23 (43) |
| No | 30 (57) |
