# Supplementary material for: Validation of the Rainbow Model of Integrated Care Measurement Tools (RMIC-MTs) in renal care for patient and care providers
Source: PLoS One. 2019 Sep 19;14(9):e0222593. doi: 10.1371/journal.pone.0222593 (PMC6752779; doi:10.1371/journal.pone.0222593)
Supplement: S3 Table — (DOCX) [file pone.0222593.s003.docx]

# Supplemental Table 3: Clarity and feasibility of the RMIC-MT patient version (n=53)

| **Characteristic** |  |
| --- | --- |
| Time (minutes) to complete questionnaire, mean (SD), range | 9 (2,7), 5-20 |
| Help to complete the questionnaire, n (%) |  |
| Yes | 7 (13) |
| No | 46 (87) |
| Difficult questions, n (%) |  |
| Yes | 1 (2) |
| No | 52 (98) |
| Upsetting questions, n (%) |  |
| Yes | NS |
| No | 53 (100) |
| Abbreviations: NS, not stated. |  |
