# Supplementary material for: Validation of the Rainbow Model of Integrated Care Measurement Tools (RMIC-MTs) in renal care for patient and care providers
Source: PLoS One. 2019 Sep 19;14(9):e0222593. doi: 10.1371/journal.pone.0222593 (PMC6752779; doi:10.1371/journal.pone.0222593)
Supplement: S4 Table — (DOCX) [file pone.0222593.s004.docx]

# Supplemental Table 4: Summary measures of the items of the RMIC-MT patient and provider version

| **RMIC-MT** | **Median** | **Mean** | **Mode** | **Standard Deviation** | **Floor effect (n, %)** | **Ceiling effect (n,%)** | **Skewness** | **Kurtosis** |
| --- | --- | --- | --- | --- | --- | --- | --- | --- |
| ***Patient version*** | |  |  |  |  |  |  |  |
| Item 1 | 4 | 4.41 | 4 | 0.65 | 98 (0.6) | 8165 (46.6) | -1.38 | 4.41 |
| Item 2 | 4 | 4.39 | 4 | 0.66 | 84 (0.5) | 8085 (46.2) | -1.31 | 3.72 |
| Item 3 | 4 | 4.35 | 4 | 0.68 | 79 (0.5) | 7659 (43.7) | -1.18 | 2.90 |
| Item 4 | 4 | 4.31 | 4 | 0.71 | 96 (0.5) | 7374 (42.1) | -1.21 | 2.71 |
| Item 5 | 5 | 4.45 | 5 | 0.64 | 77 (0.4) | 8784 (50.2) | -1.38 | 4.06 |
| Item 6 | 4 | 4.31 | 4 | 0.74 | 119 (0.7) | 7611 (43.5) | -1.32 | 2.86 |
| Item 7 | 4 | 4.38 | 5 | 0.68 | 78 (0.4) | 8210 (46.9) | -1.21 | 2.80 |
| Item 8 | 4 | 4.35 | 4 | 0.68 | 60 (0.3) | 7729 (44.1) | -1.10 | 2.32 |
| Item 9 | 4 | 4.01 | 4 | 0.93 | 270 (1.5) | 5758 (32.9) | -0.92 | 0.63 |
| Item 10 | 4 | 3.91 | 4 | 0.97 | 335 (1.9) | 5247 (30) | -0.80 | 0.24 |
| Item 11 | 4 | 4.28 | 4 | 0.71 | 84 (0.5) | 7085 (40.5) | -1.08 | 2.18 |
| Item 12 | 4 | 4.34 | 4 | 0.71 | 103 (0.6) | 7754 (44.3) | -1.24 | 2.76 |
| Item 13 | 4 | 4.41 | 5 | 0.65 | 64 (0.4) | 8430 (48.1) | -1.20 | 2.90 |
| Item 14 | 4 | 4.3 | 4 | 0.73 | 78 (0.4) | 7465 (42.6) | -1.07 | 1.82 |
| Item 15 | 4 | 3.35 | 4 | 1.28 | 1875 (10.7) | 3542 (20.2) | -0.40 | -1.00 |
| Item 16 | 4 | 4 | 4 | 0.86 | 184 (1.1) | 5324 (30.4) | -0.76 | 0.55 |
| Item 17 | 4 | 4.21 | 4 | 0.76 | 117 (0.7) | 6461 (36.9) | -1.10 | 2.03 |
| Item 18 | 4 | 4.22 | 4 | 0.79 | 150 (0.9) | 6852 (39.1) | -1.24 | 2.22 |
| Item 19 | 4 | 3.89 | 4 | 1.16 | 1026 (5.9) | 5830 (33.3) | -1.11 | 0.34 |
| Item 20 | 4 | 4.27 | 4 | 0.81 | 208 (1.2) | 7608 (43.4) | -1.43 | 2.78 |
| Item 21 | 4 | 4.38 | 5 | 0.72 | 130 (0.7) | 8338 (47.6) | -1.55 | 4.06 |
| Item 22 | 4 | 4.08 | 4 | 1.01 | 569 (3.2) | 6792 (38.8) | -1.31 | 1.38 |
| Item 23 | 4 | 4.39 | 4 | 0.68 | 89 (0.5) | 8254 (47.1) | -1.43 | 3.82 |
| Item 24 | 4 | 4.42 | 5 | 0.66 | 67 (0.4) | 8549 (48.8) | -1.28 | 3.18 |
| ***Provider version*** | |  |  |  |  |  |  |  |
| Item 1 | 5 | 4.47 | 5 | 0.74 | 81 (1.4) | 3302 (56.5) | -1.95 | 5.65 |
| Item 2 | 5 | 4.49 | 5 | 0.72 | 74 (1.3) | 3391 (58) | -2.01 | 6.08 |
| Item 3 | 4 | 4.18 | 4 | 0.85 | 71 (1.2) | 2305 (39.4) | -1.15 | 1.59 |
| Item 4 | 4 | 4.21 | 4 | 0.82 | 68 (1.2) | 2305 (39.4) | -1.19 | 1.98 |
| Item 5 | 4 | 4.26 | 4 | 0.81 | 76 (1.3) | 2506 (42.8) | -1.33 | 2.53 |
| Item 6 | 4 | 4.06 | 4 | 0.86 | 73 (1.2) | 1933 (33) | -0.88 | 0.90 |
| Item 7 | 4 | 4.04 | 4 | 0.89 | 90 (1.5) | 1923 (32.9) | -0.93 | 0.93 |
| Item 8 | 4 | 4.07 | 4 | 0.90 | 92 (1.6) | 2074 (35.5) | -0.99 | 0.97 |
| Item 9 | 4 | 3.85 | 4 | 0.95 | 113 (1.9) | 1560 (26.7) | -0.67 | 0.15 |
| Item 10 | 5 | 4.31 | 5 | 0.87 | 52 (0.9) | 3102 (53) | -1.24 | 1.19 |
| Item 11 | 4 | 4.23 | 5 | 0.90 | 63 (1.1) | 2862 (48.9) | -1.09 | 0.79 |
| Item 12 | 5 | 4.53 | 5 | 0.75 | 18 (0.3) | 3926 (67.1) | -1.59 | 2.12 |
| Item 13 | 4 | 4.21 | 5 | 0.94 | 105 (1.8) | 2882 (49.3) | -1.11 | 0.88 |
| Item 14 | 5 | 4.43 | 5 | 0.78 | 20 (0.3) | 3427 (58.9) | -1.33 | 1.44 |
| Item 15 | 4 | 3.94 | 5 | 1.29 | 555 (9.5) | 2695 (46.1) | -1.11 | 0.06 |
| Item 16 | 4 | 4.06 | 5 | 0.93 | 96 (1.6) | 2237 (38.2) | -0.87 | 0.47 |
| Item 17 | 3 | 3.36 | 4 | 1.02 | 210 (3.6) | 794 (13.6) | -0.22 | -0.52 |
| Item 18 | 3 | 3.44 | 3 | 1.01 | 172 (2.9) | 919 (15.7) | -0.23 | -0.44 |
| Item 19 | 3 | 3.26 | 3 | 1.01 | 261 (4.5) | 764 (13.1) | -0.05 | -0.35 |
| Item 20 | 4 | 3.55 | 4 | 0.96 | 150 (2.6) | 957 (16.4) | -0.35 | -0.15 |
| Item 21 | 4 | 3.61 | 4 | 0.94 | 97 (1.7) | 1068 (18.3) | -0.32 | -0.28 |
| Item 22 | 3 | 3.47 | 3 | 1.05 | 189 (3.2) | 1127 (19.3) | -0.21 | -0.57 |
| Item 23 | 4 | 3.55 | 3 | 1.06 | 186 (3.2) | 1316 (22.5) | -0.29 | -0.56 |
| Item 24 | 4 | 3.94 | 4 | 0.82 | 56 (1.0) | 1499 (25.6) | -0.50 | 0.35 |
| Item 25 | 4 | 3.61 | 3 | 0.86 | 81 (1.4) | 956 (16.3) | -0.11 | -0.05 |
| Item 26 | 4 | 3.69 | 4 | 0.92 | 122 (2.1) | 1133 (19.4) | -0.43 | 0.09 |
| Item 27 | 4 | 3.59 | 4 | 1.00 | 217 (3.7) | 1103 (18.9) | -0.48 | -0.05 |
| Item 28 | 4 | 3.54 | 3 | 0.83 | 86 (1.5) | 700 (12) | -0.18 | 0.23 |
| Item 29 | 4 | 3.64 | 4 | 0.86 | 92 (1.6) | 860 (14.7) | -0.39 | 0.24 |
| Item 30 | 4 | 3.65 | 4 | 0.84 | 74 (1.3) | 859 (14.7) | -0.33 | 0.23 |
| Item 31 | 5 | 4.44 | 5 | 0.77 | 27 (0.5) | 3350 (57.3) | -1.47 | 2.33 |
| Item 32 | 5 | 4.37 | 5 | 0.82 | 30 (0.5) | 3151 (53.9) | -1.40 | 1.83 |
| Item 33 | 5 | 4.39 | 5 | 0.79 | 26 (0.4) | 3191 (54.6) | -1.26 | 1.43 |
| Item 34 | 5 | 4.36 | 5 | 0.80 | 29 (0.5) | 3088 (52.8) | -1.20 | 1.25 |
| Item 35 | 5 | 4.33 | 5 | 0.84 | 30 (0.5) | 3104 (53.1) | -1.10 | 0.72 |
| Item 36 | 4 | 3.78 | 5 | 1.19 | 418 (7.1) | 2080 (35.6) | -0.75 | -0.18 |
| Item 37 | 4 | 3.52 | 3 | 1.13 | 437 (7.5) | 1332 (22.8) | -0.48 | -0.30 |
| Item 38 | 3 | 3.18 | 3 | 1.18 | 758 (13) | 860 (14.7) | -0.28 | -0.53 |
| Item 39 | 3 | 3.47 | 3 | 1.05 | 358 (6.1) | 1043 (17.8) | -0.41 | -0.02 |
| Item 40 | 3 | 3.49 | 3 | 1.09 | 396 (6.8) | 1209 (20.7) | -0.42 | -0.14 |
| Item 41 | 4 | 4.22 | 5 | 0.93 | 94 (1.6) | 2675 (45.7) | -1.42 | 1.81 |
| Item 42 | 4 | 3.55 | 4 | 1.23 | 429 (7.3) | 1378 (23.6) | -0.62 | -0.72 |
| Item 43 | 5 | 4.3 | 5 | 0.91 | 76 (1.3) | 2992 (51.2) | -1.50 | 2.03 |
| Item 44 | 5 | 4.31 | 5 | 0.88 | 62 (1.1) | 2999 (51.3) | -1.48 | 2.09 |
| Item 45 | 4 | 4.21 | 5 | 0.95 | 88 (1.5) | 2698 (46.1) | -1.36 | 1.45 |
| Item 46 | 4 | 4.17 | 5 | 1.00 | 137 (2.3) | 2689 (46) | -1.30 | 1.21 |
| Item 47 | 4 | 4.15 | 5 | 0.95 | 112 (1.9) | 2547 (43.5) | -1.17 | 1.15 |
| Item 48 | 4 | 4.27 | 5 | 0.83 | 53 (0.9) | 2692 (46) | -1.21 | 1.64 |
