# Supplementary material for: Validation of the Rainbow Model of Integrated Care Measurement Tools (RMIC-MTs) in renal care for patient and care providers
Source: PLoS One. 2019 Sep 19;14(9):e0222593. doi: 10.1371/journal.pone.0222593 (PMC6752779; doi:10.1371/journal.pone.0222593)
Supplement: S5 Table — (DOCX) [file pone.0222593.s005.docx]

# Supplemental Table 5: Descriptive statistics and internal consistency RMIC-MT patient version

| **Scale/ item** | **Mean score (SD)** | **% nE^a^** | **ITC^b^** | **Cronbach's alpha** |
| --- | --- | --- | --- | --- |
| **Clinical coordination** | 4,37 (0,58) |  |  | 0,93 |
| Listening |  | 1,8 | 0,81 |  |
| Preference integration |  | 1,9 | 0,82 |  |
| Questioning |  | 1,5 | 0,78 |  |
| Communicating |  | 2,4 | 0,79 |  |
| Explaining |  | 1,7 | 0,77 |  |
| Shared decision-making |  | 3 | 0,77 |  |
| **Professional coordination** | 4,18 (0,66) |  |  | 0,86 |
| Interdisciplinary information continuity |  | 3,2 | 0,67 |  |
| Interdisciplinary treatment continuity |  | 4,3 | 0,68 |  |
| Interdisciplinary contact |  | 5 | 0,65 |  |
| Interdisciplinary coordination |  | 2,2 | 0,73 |  |
| **Organisational coordination** | 4,37 (0,59) |  |  | 0,84 |
| Time management |  | 2,4 | 0,73 |  |
| Appointments |  | 4,5 | 0,63 |  |
| Results |  | 2,9 | 0,67 |  |
| Accessibility |  | 1,6 | 0,69 |  |
| **Person-centeredness** | 3,96 (0,89) |  |  | 0,87 |
| Family circumstances |  | 7,5 | 0,77 |  |
| Social circumstances |  | 9,5 | 0,77 |  |
| **Overall care coordination** | 4,27 (0,54) |  |  | 0,94 |
| a nE = the proportion of negative experiences , in % | |  |  |  |
| b Corrected item-total correlation (ITC) within a domain are shown. | | |  |  |
