# Supplementary material for: Validation of the Rainbow Model of Integrated Care Measurement Tools (RMIC-MTs) in renal care for patient and care providers
Source: PLoS One. 2019 Sep 19;14(9):e0222593. doi: 10.1371/journal.pone.0222593 (PMC6752779; doi:10.1371/journal.pone.0222593)
Supplement: S6 Table — (DOCX) [file pone.0222593.s006.docx]

# Supplemental Table 6: Descriptive statistics and internal consistency RMIC-MT provider version

| **Scale/ item** | **Mean score (SD)** | **% nEa** | **ITCb** | **Cronbach's alpha** |
| --- | --- | --- | --- | --- |
| **Cultural competence** | 4.26 (0.80) |  |  | 0,90 |
| Support |  | 8,9 | 0,78 |  |
| Teamwork |  | 7,4 | 0,77 |  |
| Respect |  | 6,2 | 0,76 |  |
| Fellowship |  | 8,3 | 0,76 |  |
| **Person-centeredness** | 4,32 (0,67) |  |  | 0,90 |
| Listening |  | 2,3 | 0,78 |  |
| Interpersonal trust |  | 2,3 | 0,74 |  |
| Questioning |  | 3,4 | 0,80 |  |
| Preference integration |  | 3,8 | 0,78 |  |
| Social circumstances |  | 4,6 | 0,72 |  |
| **Technical competence** | 3.41 (0.91) |  |  | 0,84 |
| Interoperable EHRs |  | 22,1 | 0,72 |  |
| Interoperable IT tools |  | 15,4 | 0,66 |  |
| Data integration |  | 12,2 | 0,68 |  |
| Outcome transparency |  | 12,6 | 0,63 |  |
| **Professional coordination** | 3.42 (0.83) |  |  | 0,87 |
| Interdisciplinary fragmentation |  | 16,9 | 0,72 |  |
| Interdisciplinary teamwork |  | 15,4 | 0,71 |  |
| Interdisciplinary follow-up |  | 17 | 0,72 |  |
| Interdisciplinary communication |  | 20,2 | 0,67 |  |
| Interdisciplinary coordination |  | 20,3 | 0,64 |  |
| **Clinical coordination** | 4.35 (0.67) |  |  | 0,77 |
| Follow-up of care |  | 4,3 | 0,66 |  |
| Case management |  | 4 | 0,64 |  |
| Shared decision-making |  | 1,5 | 0,52 |  |
| **Triple aim** | 4.37 (0.68) |  |  | 0,9 |
| Monitoring & follow-up |  | 2,6 | 0,80 |  |
| Quality objectives |  | 2,4 | 0,79 |  |
| Needs assessment |  | 2,8 | 0,75 |  |
| Outcome assessment |  | 2,7 | 0,73 |  |
| Experience assessment |  | 4,2 | 0,74 |  |
| **Organisational coordination** | 3.63 (0.82) |  |  | 0,85 |
| Inter-organisational resources |  | 7,9 | 0,68 |  |
| Inter-organisational staff |  | 11,9 | 0,78 |  |
| Inter-organisational coordination |  | 6,1 | 0,72 |  |
| **System coordination** | 3.61 (0.78) |  |  | 0,9 |
| Interdisicplinary incentives |  | 7,5 | 0,87 |  |
| Care coordination incentives |  | 6,4 | 0,84 |  |
| Inter-organisational incentives |  | 7,1 | 0,80 |  |
| **Community centeredness** | 4,00 (0,79) |  |  | 0,89 |
| Health promotion |  | 5,3 | 0,80 |  |
| Population needs |  | 8,5 | 0,76 |  |
| Community partnerships |  | 4,5 | 0,76 |  |
| Community collaboration |  | 5,8 | 0,75 |  |
| **Overall care coordination** | 3.95 (0.49) |  |  | 0,93 |
| a nE = the proportion of negative experiences , in % |  |  |  |  |
| b Corrected item-total correlation (ITC) within a domain are shown. | |  |  |  |
